# The Consumption of Edible Leaves by Afro-Descendants in French Guiana and Suriname: An Overview of a Constantly Evolving Ethno-Culinary Practice

**DOI:** 10.3390/plants15132096

**Published:** 2026-07-06

**Authors:** Marc-Alexandre Tareau, Alexander M. Greene, Clarisse Ansoe-Tareau, Nicholaas Pinas, Michael Rapinski

**Affiliations:** 1 Institut Santé des Populations d’Amazonie, UA 17 Laboratory, CHU de Guyane, 97300 Cayenne, France; 2 MELISSE NGO, 97300 Cayenne, France; ansoe.clarissemana@gmail.com; 3 LEEISA, UAR 3456, 97300 Cayenne, France; accidentalshrike@gmail.com (A.M.G.); michael.rapinski@umontreal.ca (M.R.); 4 Naturalis Biodiversity Center, 2333 Leiden, The Netherlands; nicholaas.pinas@hotmail.com; 5 Eco-Anthropologie, UMR 7206, 75016 Paris, France

**Keywords:** food heritage, Guiana Shield, food systems, ethnobotany, leafy greens, callaloo, unseen foods, interculturality

## Abstract

This paper explores the culinary and cultural significance of cooked leafy vegetables among Afro-descendant communities in French Guiana and Suriname, including French Guianese and Surinamese Creoles, Maroons, and Haitian migrants. While leafy greens play a major dietary role across sub-Saharan Africa, their consumption in the Americas remains understudied. This ethnobotanical study of edible leafy plants is based on surveys of local markets, gardens and residents. Drawing on 26 informal interviews conducted in four local languages (French, French Guianese Creole, Haitian Creole, and Nengee Tongo), we describe 36 species of edible leaves from 20 plant families consumed in the region. Our findings show that although the practice of eating leafy greens is widely shared, the species selected, their names, and their perceived properties vary noticeably across cultural groups. Some plants are eaten exclusively by Maroons (e.g., *Cestrum latifolium*, *Capsicum* spp.), others by Haitians (e.g., *Corchorus olitorius*, *Rivina humilis*), and some have fallen into disuse among younger generations. These differences are shaped by ecological availability, cultural memory, food-medicine beliefs, and interethnic influences. We suggest that the term *callaloo* (referring to both dishes and leafy vegetables), which circulates in multiple linguistic and culinary forms throughout the African diaspora, can serve as a metaphor for the interculturalization of foodways. More than ingredients, these leafy vegetables act as dynamic cultural markers—symbols of resilience, transmission, and transformation. In a context of rapid globalization, where unseen foods risk sinking further into obscurity, these plant-based traditions highlight both the adaptability and fragility of Afro-descendant culinary heritage in the Guiana Shield.

## 1. Introduction

Leafy vegetables play a particularly important role in the diet of sub-Saharan Africa [1,2,3]. According to Walker [4], no other culinary tradition occupies such a central place in the cuisines of this culturally diverse region. Many African leafy greens impart a bitter flavor, a common feature of African dishes, and one whose biocultural importance in West and Central Africa has been highlighted in the literature [5,6]. Some leaves with mucilaginous properties are used as binding agents in sauces or to thicken stews, notably okra (*Abelmoschus esculentus* (L.) Moench), roselle (*Hibiscus sabdariffa* L.), baobab (*Adansonia digitata* L.) and jute mallow (*Corchorus olitorius* L.) [7]. Certain domesticated or semi-domesticated species are grown in home gardens (e.g., okra), while many others are weeds or harvested in the wild (e.g., baobab leaves) [8].

Unsurprisingly, the African tropism for leafy vegetables can also be found among Afro-descendant populations in the Americas, many of whom consume cooked leaves as a complement to rice or meat or as a dish in their own right [9,10]. In this paper, we use the term “leafy vegetables” to refer to edible leaves consumed when cooked, although we also employ common synonyms such as “edible leaves”, “potherbs” or “leafy greens” interchangeably. Despite their diversity of flavors and textures, these leafy vegetables are generally prepared in a similar manner: they are chopped and then cooked in oil or water, with or without meat or other seasonings. Falling clearly within the food-medicine continuum [11] of Afro-descendant communities, these leafy vegetables are considered to have a definite medicinal value among these populations. Within the Creole community of French Guiana, for instance, they are said to ‘cleanse the blood’, to be ‘refreshing’, and to act as vermifuges [12]. Numerous nutritional studies have shown that leafy greens are rich in macronutrients (e.g., proteins and amino acids) and micronutrients (e.g., minerals and vitamins A, C and E), i.e., Traoré et al. [3]. Although nutrient-dense, many leafy green vegetables continue to be ‘unseen foods’, which are “deeply embedded in local socio-cultural settings—shaped by ecological knowledge, identity, heritage, belief systems, practices, and community-based economies” yet operate outside “formalized and regulated socio-economic frameworks” [13]. Because of their cultural embeddedness, such unseen foods hold considerable potential to address hidden hunger, improve diet quality and contribute to food security in marginalized communities and populations [14,15,16].

Considering that leafy green vegetables are often central to culinary and medicinal traditions, they represent valuable indicators of both biocultural knowledge and local food systems. Their persistence in the collective memory and practices of Afro-descendant communities therefore offers a lens to explore food heritage, knowledge transmission, and cultural continuity in the context of colonial legacies. Despite the rich literature on the culinary, nutritional, and medicinal roles of leafy vegetables in Africa and the African diaspora, little is known about how these practices have been maintained, transformed, or diversified among Afro-descendant populations in the Guianas. Fleury [17] and Bilby et al. [18] have conducted extensive surveys of leafy vegetables consumed among the Aluku and Ndjuka Maroon (or Bushinenge) Afro-descendant groups in French Guiana. Kinupp and Lorenzi [19,20] have also conducted an extensive survey of leafy green vegetables consumed in Brazil. Although many of the species treated by Kinupp and Lorenzi occur in the Guianas, these authors do not systematically attribute their usage to specific cultural groups. Such studies have generally focused on single cultural groups or local contexts, thus providing a limited understanding of the circulation and consumption of leafy vegetables across culturally distinct Afro-descendant groups in the region. Food practices within diasporic societies may reflect broader processes of cultural transmission, adaptation, and creolization, and the tropism for leafy vegetables constitutes a quintessential example of biocultural heritage among the African diaspora.

Beyond their cultural importance and high nutrient density, leafy vegetables may contribute to sustainable food systems because they are locally available and well-adapted to tropical environments. Moreover, rapid demographic changes linked to migration, particularly the arrival of Haitian migrants in French Guiana, further contribute to the diversification and transformation of plant-based food traditions and heritage in the region. To our knowledge, no study has yet compared the diversity and use of edible leafy plants across multiple Afro-descendant communities in the Guiana Shield. To address this knowledge gap, the present study aims to (1) examine the diversity of leafy vegetables currently consumed by various Afro-descendant communities in French Guiana and Suriname, (2) compare the overlaps and differences in species selection among these groups, and (3) assess the extent to which these practices reflect continuity with African and Afro-American food traditions. It is hypothesized that shared species will emerge across cultural groups, reflecting processes of intercultural exchange and creolization. In addition, certain species will remain culturally specific, reflecting distinct cultural histories and knowledge transmission pathways. By bringing together data from several cultural groups, this research provides the first comparative ethnobotanical inventory of culturally significant leafy vegetables among Afro-descendant groups in the Guiana Shield and sheds light on the dynamics of food heritage in the region.

Beyond its ethnobotanical and anthropological contributions, this study is also relevant for plant science, as it documents a diverse group of edible leafy species (many of which remain underutilized or poorly studied) and highlights their potential importance for nutrition, agrobiodiversity, and sustainable food systems.

## 2. Results

### 2.1. Ethnobotanical Results

**Figure 1:**
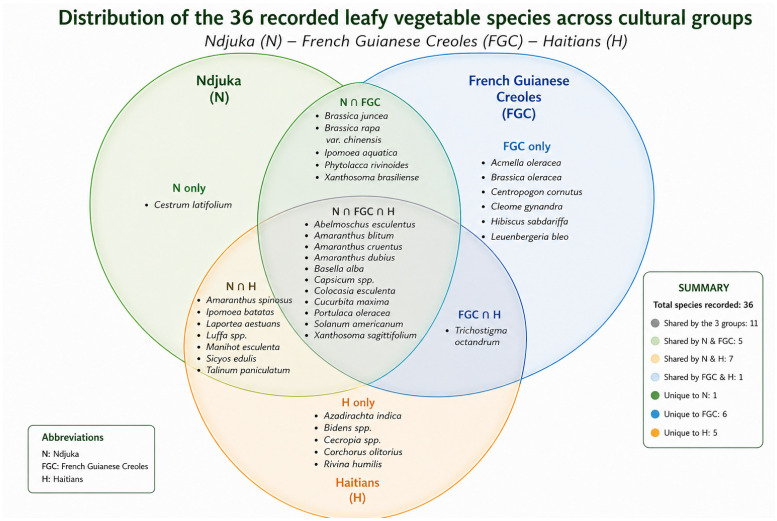
Venn diagram showing the distribution of the 36 recorded leafy vegetable species across three Afro-descendant cultural groups: Ndjuka (N), French Guianese Creoles (FGC), and Haitians (H). Species are classified according to whether they were cited by a single group (unique), shared between two groups, or common to all three groups (central intersection).

A total of 36 species from 20 botanical families were recorded as edible leafy vegetables in the study area (Table 1). Beyond this diversity, the results reveal distinct patterns of plant use across cultural groups, characterized by both shared and group-specific species (Figure 1).

As shown in Figure 1, several widely consumed species are shared across all Afro-descendant communities, including *Amaranthus* spp., *Basella alba*, and *Ipomoea batatas*. These plants, commonly found in markets and home gardens, reflect a shared culinary base and likely result from long-term processes of intercultural exchange.

In contrast, some species appear to be strongly associated with specific cultural groups. For instance, *Corchorus olitorius*, *Rivina humilis*, and *Trichostigma octandrum* are primarily consumed by Haitian communities, while *Cestrum latifolium* and *Capsicum* spp. are mainly associated with Maroon (Ndjuka) populations. French Guianese Creoles, in turn, reported the use of species such as *Centropogon cornutus* and *Hibiscus sabdariffa*, some of which appear to be declining in use.

These patterns highlight both continuity and divergence in plant use, reflecting processes of knowledge transmission, adaptation, and intercultural exchange. They also illustrate the dynamic nature of Afro-descendant food systems in the Guiana Shield, shaped by ongoing processes of interculturalization. The detailed species-level descriptions that follow should be read in light of this comparative framework, highlighting both shared patterns and culturally specific practices.

### 2.2. Species Mentioned During the Survey

#### 2.2.1. Amaranthaceae

Several species belonging to the genus *Amaranthus* are consumed by all surveyed communities. Cultivated species (which are also sometimes collected from disturbed environments, where they grow spontaneously) like *Amaranthus dubius* Mart. ex Thell. or *Amaranthus cruentus* L. are often found in markets in the form of bunches of the leafy stems. These species are commonly called *zepina* by French Guianese and Haitian Creoles and *kalalu* by Ndjuka Maroons, with the exception of *Amaranthus blitum* L., which has smaller leaves and is called *mboya* by Ndjuka Maroons. A related species, *Amaranthus spinosus* L., called *zepina kochon* (“pig spinach”) in Creole, is rarely sold in markets but is sometimes used in rural areas to garnish soups. All these species apparently originate from Central and South America [21], although they have been introduced and naturalized in Africa, Asia and Europe [22].

**Figure 2:**
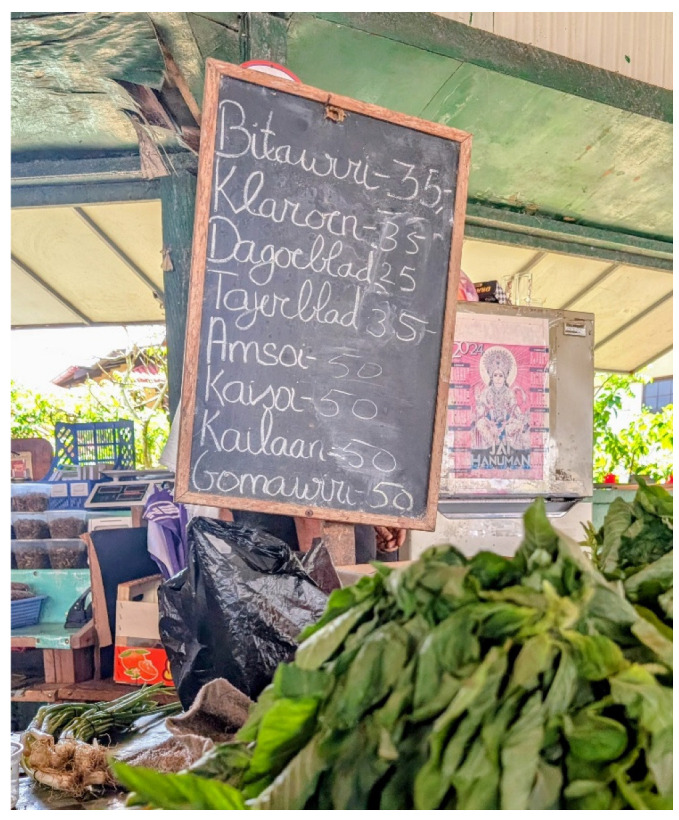
Chalkboard listing various leafy vegetables sold at a market stall in Paramaribo (Suriname), illustrating the vernacular names, in Sranan Tongo, of edible greens commonly sold in local markets. From top to bottom on the chalkboard: *bita wiri* (*Cestrum latifolium*), *klaroen* (*Amaranthus* spp.), *dagoe blad* (*Ipomoea aquatica*), *tajer blad* (*Colocasia esculenta* or *Xanthosoma sagittifolium*), *amsoi* (*Brassica juncea*), *kaisoi* (probably a *Brassica juncea* variety), *kailaan* (probably a *Brassica oleracea* variety), *goma wiri* (*Solanum americanum*). Photo credit: Guillaume Odonne, 2025.

One of the most widely consumed leafy greens in sub-Saharan Africa is red amaranth, *A. cruentus* [23]. *A. dubius* and *A. blitum* are also important vegetables in Africa, and are found both in the wild and in cultivation [2]. In Jamaica, *Amaranthus viridis* L., known as *callaloo*, is the most widely consumed leafy vegetable [24] and is cultivated in large quantities and marketed in canned form, notably for export to the USA [10]. In Suriname, *Amaranthus* spp. are cultivated as vegetables and sold in Paramaribo markets (Figure 2).

No species of the genus *Celosia* were mentioned during the survey, although they are present in French Guiana, are used medicinally by some communities, and are consumed as a potherb in West Africa [10].

#### 2.2.2. Araceae

**Figure 3:**
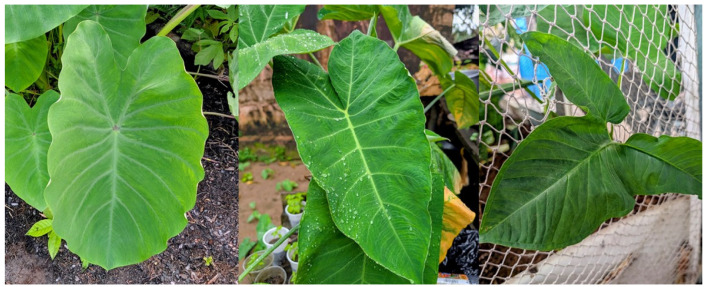
From left to right, leaves of *Colocasia esculenta*, *Xanthosoma sagittifolium* and *Xanthosoma brasiliense*, edible when prepared as cooked greens in French Guiana and Suriname. Photo credits: Marc-Alexandre Tareau, 2025.

The cooked leaves of *Colocasia esculenta* (L.) Schott and *Xanthosoma sagittifolium* (L.) Schott (Figure 3) are a staple of the Maroon diet [18]. The aerial parts of these two species (called *taya uwii* in Nenge Tongo) are only edible when cooked because of the presence of irritating oxalate crystals [25]. The young, tender leaves are generally preferred, and the purple-stemmed variety previously called *Xanthosoma violaceum* (now a synonym of *X. sagittifolium* [26,27] is particularly appreciated. These species are apparently called *chou* (“cabbage “) in Creole, in reference to the leaves being eaten cooked. It should be noted that in Québec, Canada, another plant in the Araceae family, *Symplocarpus foetidus* (L.) W. Salisb., whose leaves are edible, is also called *chou puant* (“skunk cabbage”), likely for the same reasons as the consumption of the young leaves is attested, namely among the Iroquois and Senaca Indigenous First Nation people [28,29].

The terms *chou de Chine*, *dachin* and *sineisi taya*, designating *Colocasia esculenta* varieties, mean ‘Chinese cabbage’ because these plants were introduced from Asia to the Americas in the 18th century, where they were adopted by local populations. An opposite case are *Xanthosoma* species (the taniers), which are of American origin and were widely consumed by pre-Columbian populations of Central America and the Caribbean. These species are called ‘chou caraïbes’ (“Caribbean cabbage”) in French in reference to their geographical origin. Names like *yautia*, *taya* or *malanga* are of respectively Taino, Carib, and Arawak origin [30,31,32], and are the etymological basis of the English word “tanier”, as well as *tayove* and *taioba* [32,33]. During the second half of the 18th century, Chevalier de Préfontaine [33] (pp. 14,17) wrote: “*Tayove. Corrupted form of the Indian word taya. This plant is known in the islands as ‘Chou Caraïbes’. They [the leaves] are very good in soup, and they are used in Calalou*.” (Translation by the authors of the following original quotation: “*Tayove. Nom corrompu du mot indien* taya*. Cette plante est connue dans les isles sous le nom de chou caraïbe. […] Elles [les feuilles] sont très bonnes dans la soupe*: *elles entrent dans le* Calalou.”) Later, Descourtilz [34] (p. 5) noted that “*The leaves* [of “Caribbean cabbage”] *are eaten in soup like those of ordinary cabbage*.” (Translation by the authors of the following original quotation: “*Ses feuilles se mangent dans la soupe comme celles du Chou ordinaire*.”)

*Xanthosoma brasiliense* (Desf.) Engl. is another species cultivated for its leaves (unlike *C. esculenta* and *X. sagittifolium,* its roots are not consumed in French Guiana). Maroons call it *butter blad* in Sranan tongo (“butter leaf”, because it is so tender) and Creoles from the West Indies call it *zerbaj* [35], *herbage* and *herbe à calalou* [26]. The name *siguine* is also sometimes used in the West Indies, probably because the leaves resemble wild *Philodendron* species, which are more commonly known by this vernacular name. As early as 1910, Haudricourt [36] (p. 54) wrote that: “*The plant is cultivated solely for its leaves. […] These cooked leavess have a pronounced and very delicate flavor. This plant is known in the other West Indies as “Indian Kale”; it is to this plant that the name “chou caraïbe” was first applied; it is currently known in Guadeloupe as Z’herbe à calalou (calalou herb), due to its use in the stew called calalou*.” (Translation by the authors of the following original quotation: “[…] *la plante est uniquement cultivée pour ses feuilles. […] Ces feuilles cuites ont une saveur prononcée et très délicate. Cette plante est connue dans les autres Antilles sous le nom d’*Indian kale *(Chou indien); c’est à elle qu’à dû s’appliquer d’abord le nom de Chou caraïbe; actuellement elle est connue à la Guadeloupe sous le nom de* Z’herbe à calalou *par suite de son utilisation dans le ragout appelée* Calalou.”) On the other hand, it appears that the leaves of *Xanthosoma undipes* (K. Koch & C.D. Bouché) K. Koch (Barabé and Gibernau [26] include this species within *X. sagittifolium.*), which also grows in French Guiana (*pon taya* in Nengee Tongo and *gro tayov* or *tadjo* in French Guianese Creole) are not consumed, since they were not reported during the survey.

There is considerable confusion surrounding the morphological similarity between these species and their numerous cultivars [37], as well as the fact that they are sometimes referred to by the same name by local populations [38]. For example, although they are both called *taya* in Nengee Tongo, only the leaves of the *eddoe* variety of taro (*Colocasia esculenta* var. *antiquorum*) are eaten, while those of the dasheen (*Colocasia esculenta* var. *esculenta*) variety are rarely eaten by Maroons. Finally, our data suggest that populations of African descent do not consume the leaves of *Caladium* and *Alocasia* species. *Caladium bicolor* (Aiton) Vent. leaves and flowers are sold in Paramaribo markets but appear to be consumed only by the Hindustani population.

#### 2.2.3. Asteraceae

*Acmella oleracea* (L.) R.K. Jansen is an annual plant called *jambu* in Brazil (Para cress in English), which is native to South America and produces a numbing or anesthetic effect in the mouth when eaten. This species is widely consumed in the Indian Ocean region and in Southeast Asia. Both fresh and cooked leaves are used in dishes such as the soup *tacacá* in northern Brazil [20]. This same dish is called *brèdes mafane* in Saint-Georges-de-l’Oyapock, a border town situated across the river from the Brazilian state of Amapá. The use of this name is surprising, as it is the term used to designate this species in Madagascar, where it is a main ingredient in the island’s national dish, *romazava* [39]. Moreover, leafy vegetables are called *brèdes* in the Mascarene Islands. This term probably derives from the Latin word *blitum*, which is also the source of the French ethno-taxons *blette* (for *Amaranthus blitum* L.) and *bette* (*Beta vulgaris* L.) and the English beet [40].

A few Brazilian vendors sell the leaves in the Cayenne market, but they are not widely consumed outside the Brazilian community at the moment. One Haitian vendor who did not know how to prepare it reportedly sold this plant to the Brazilian and Javanese community, an indication that the plant is undergoing cross-cultural exchange. The biogeographical history of this species warrants further investigation, as it is mainly cultivated and consumed in two geographically distant regions of the world: the mouth of the Amazon, and islands in the Indian Ocean.

A Haitian man reported that *Bidens* spp. leaves were sometimes eaten cooked with rice in rural Haiti. However, this use does not appear to have spread to French Guiana, although species from this genus (primarily *Bidens cynapiifolia* Kunth and *Bidens pilosa* L.) are common in disturbed areas. A Creole woman claimed to have consumed the leaves of this plant in 24 h dietary recalls in Guadeloupe [41]. Its use thus appears to be limited or marginal among Afro-descendant communities of the region, and is likely due to influence from Brazilian or other communities where its use is more common.

#### 2.2.4. Baselaceae

Malabar spinach (*Basella alba* L.) is a perennial climbing plant native to India that has spread rapidly throughout tropical Africa and the Americas [42]. This leafy vegetable has green or red climbing stems and is grown for its succulent spinach-like leaves. In some parts of tropical Africa, Malabar spinach has become an integral part of traditional diets [43]. This plant’s vernacular names, *spenasi* in Nengee Tongo and *zepina* in Creole, derive from the English word “spinach” and its French translation “*épinard*”.

In French Guiana, *Basella alba* L. is cultivated and common in farmers markets, convenience stores and supermarkets. This species is described as a “regularly consumed spinach” in some publications [44]. It is also available in markets in Paramaribo, although it appears to be less popular there.

#### 2.2.5. Brassicaceae

**Figure 4:**
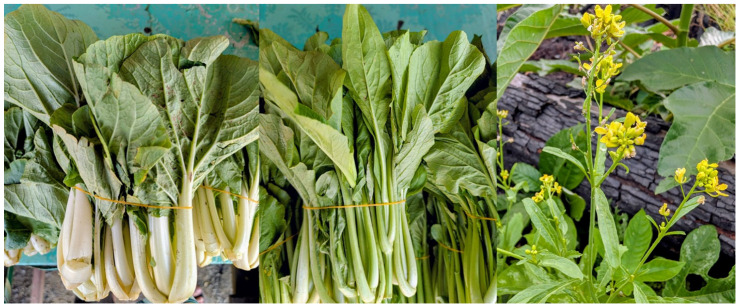
Leaves of *Brassica rapa* var. *chinensis* (**left**) and leaves (**center**) and flowers (**right**) of *Brassica juncea*, all prepared as cooked greens in French Guiana and Suriname. Photo credits: Marc-Alexandre Tareau, 2025.

Several species of cultivated Brassicaceae are widely consumed in French Guiana. In Nenge Tongo and Sranan Tongo, *Brassica rapa* var. *chinensis* (*pakchoi* or *bok choi*) and *Brassica juncea* (L.) Czern. (*amsoi* or *chap soi*; Figure 4) retain their Chinese names. Both species originated in southern China and were introduced to the Americas by Chinese immigrants [45]. Often confused with each other, these leafy vegetables are called *chou chinwa* in French Guianese Creole, meaning “Chinese cabbage”. These species are also widely present in markets in Surinam, where there is an important Chinese community.

Another European species, *Brassica oleracea* L., is grown and eaten mainly by the Brazilian community, who call it *kale* or *couve*. The importance of this vegetable in Brazil is no doubt a legacy of European immigration to the country over recent centuries. This was the only leafy vegetable found at the market of Saint-Georges-de-l’Oyapock, where an important Brazilian population resides. In the US, where it was commonly fed to slaves during the colonial era, the variety of this plant called “collard greens” is central to southern African American culture and identity [46,47].

#### 2.2.6. Cactaceae

**Figure 5:**
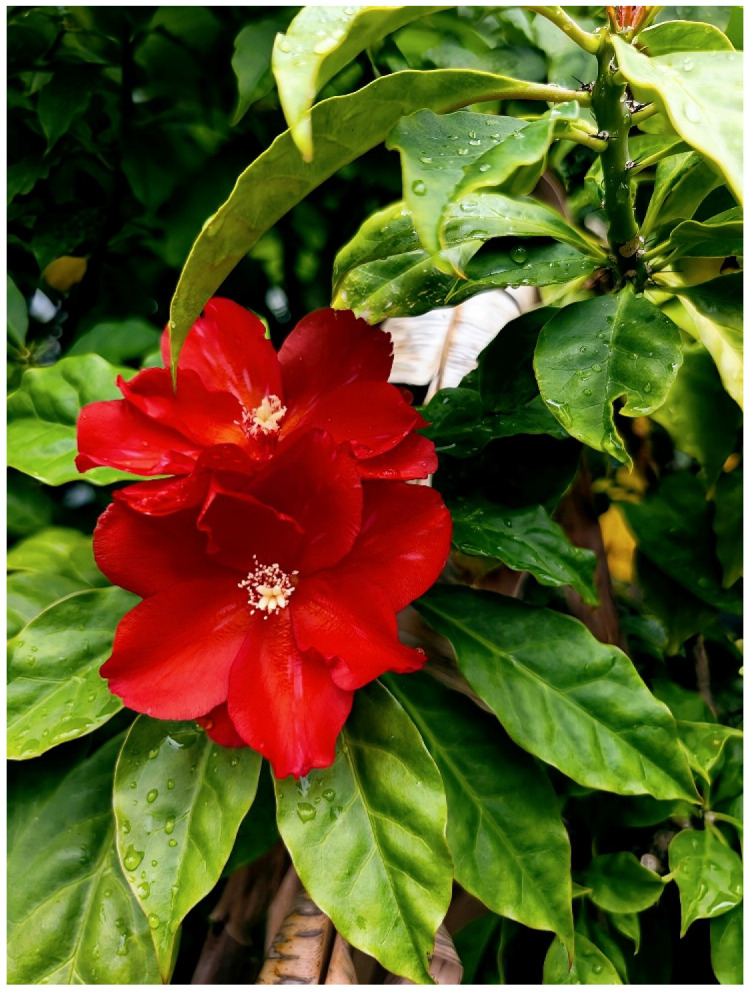
Leaves and flowers of *Leuenbergeria bleo*, photographed in a garden of Cayenne, the capital city of French Guiana. Photo credit: Marc-Alexandre Tareau, 2025.

During the survey, only one species of cactus was mentioned: the cultivated *Leuenbergeria bleo* (Kunth) Lodé., which was called *fey pereskya* (Figure 5). A French Guianese Creole man told us that it can be eaten raw or cooked, and that its texture is slightly slimy. He said that he had seen this use described in a book about edible leaves. Although we know that some people consume the leaves of species in the genera *Cereus* and *Opuntia*, these were not mentioned during this study. This is probably because their succulent form means that they are not usually considered to be leaves.

#### 2.2.7. Campanulaceae

The only species of this family mentioned during the survey was *Centropogon cornutus* (L.) Druce, a wild-growing species found in the secondary forests of coastal French Guiana. Known as *radyé pété* (literally ‘fart grass’) in French Guianese Creole, it was once added to some Creole dishes. This species was not found in local markets and it seems to have fallen out of use, since it was only cited by one person:

*“Anvan, asou bitasyon, nou té ka servi sa bokou. Nou té ka mété’l annan kalalou a, annan bouyon wara a…Men moun aprézan pa konnet sa.”* [In the countryside, this leaf (*C. cornatus*) was used a lot. It was added to *kalalou* and *bouyon wara* (Creole dishes)…But people nowadays don’t know about it anymore.]”

French Guianese woman, 83 old years, living in Cayenne.

#### 2.2.8. Cleomaceae

According to our interviews, *Cleome gynandra* L. species, called *mouzambe* in French Guianese Creole, was once eaten by French Guianese Creoles. This use also appears to have nearly disappeared, given that only one person mentioned it. Descourtilz [34] mentions this plant being eaten raw or cooked in the West Indies at the time of his inventory in the early 19th century. He suggests that it is native to Africa, where it is still used as a leafy vegetable throughout the continent [48], since its common name in French Guianese Creole, *mouzambe*, is borrowed directly from Bantu languages. Tinde van Andel et al. [49] (p. 855) also noted that “*the African herb Cleome gynandra was commonly grown and eaten raw or cooked like spinach in 18th-century Suriname. This plant is not eaten anymore, but is naturalized as a weed in Suriname and French Guiana*.”

#### 2.2.9. Convolvulaceae

**Figure 6:**
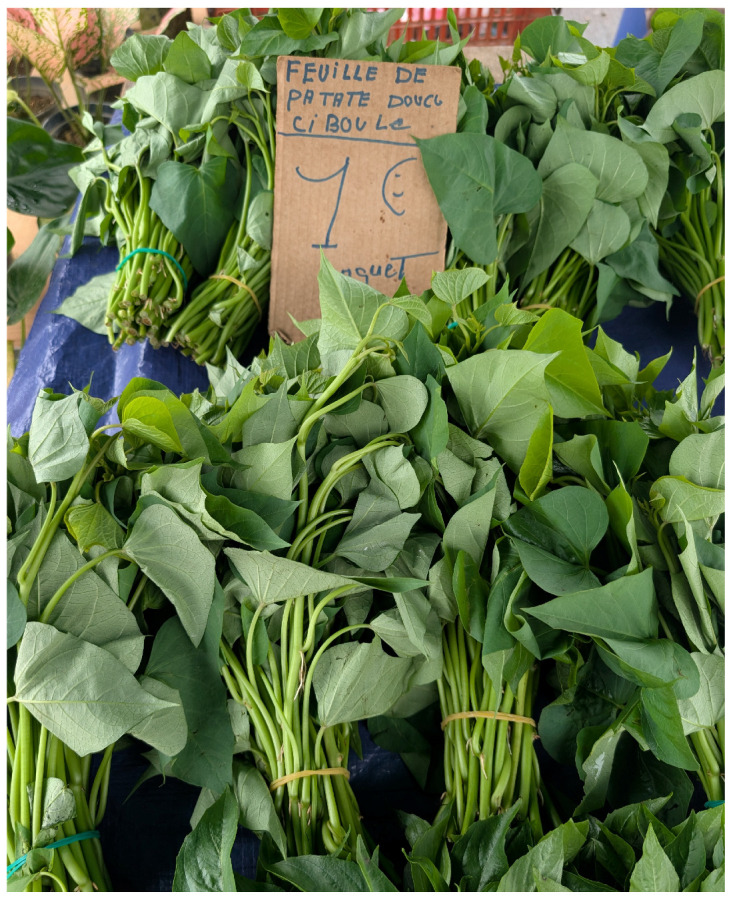
Bundles of young sweet potato (*Ipomoea batatas*) shoots sold at a market in Cayenne. From top to bottom on the price sign: *feuille de patate douce* (leaves of *Ipomoea batatas*), *ciboule* (*Allium fistulosum* L.) Photo credit: Marc-Alexandre Tareau, 2025.

Young sweet potato leaves, *Ipomoea batatas* (L.) Lam., are occasionally eaten by Creole and Maroon populations (Figure 6). However, the leaves of water spinach, *Ipomoea aquatica* Forssk., are far more commonly used as a leafy green. This species, which is native to Africa and Asia, is grown exclusively for this purpose and is increasingly found in French Guianese markets, often cultivated by Hmong farmers. There are two main varieties: one with large leaves and another with smaller leaves. Both are known as *dagoe blad,* literally ‘dog’s leaf’ in Sranan Tongo (Figure 2), because they grow spontaneously in canals along roadsides where stray dogs walk.

#### 2.2.10. Cucurbitaceae

The use of *Luffa* spp., *Cucurbita maxima* Duchesne and *Sicyos edulis* Jacq. by French Guianese and Haitian Creoles is generally restricted to the fruits of these species, of which the latter two can be bought in local markets and supermarkets throughout French Guiana. However, an elderly French Guianese Creole woman mentioned that sprouts and leaves (specified by the word *fey*) of *C. maxima*, *Luffa* spp., and *S. edulis* were previously used by French Guianese Creoles to make broths. Since no one mentioned currently consuming these species, their use appear to be disappearing among Afro-descendants in French Guiana. However, Hmong people in French Guiana continue to consume several of these greens in French Guiana (com. pers., A. Greene), which could eventually lead to their readoption by Afro-descendant populations.

#### 2.2.11. Euphorbiaceae

Cassava (*Manihot esculenta* Crantz) leaves (*maniva* in Brazilian Portuguese) are consumed by the Brazilian community in French Guiana, where they are integral to *maniçoba*, a festive dish from Parà state. This dish can be found at festive events throughout French Guiana, where street vendors sell it in areas with large Brazilian populations, such as Saint-Georges-de-l’Oyapock. Although the tuberous roots of cassava are a staple in the diets of virtually all Afro-descendant and Indigenous peoples throughout French Guiana [50,51,52,53,54,55], none of these cultural communities report eating cassava leaves. Even among the Palikur—an Indigenous people living on both sides of the Franco-Brazilian border, for whom cassava is the dietary staple [53,55]—the leaves are not eaten, despite awareness of their culinary use in the Brazilian community (com. pers., Michael Rapinski).

Through cross-cultural diffusion, it is likely that the consumption of cassava leaves as a potherb will gradually spread to other communities. As evidence that such diffusion is already underway, one Haitian vendor who sells cassava leaves to Brazilian and African customers in Cayenne explained that some members of the Haitian community have begun incorporating this plant into leafy dishes. Another Haitian man reported that this culinary practice is also found in Haiti. In another example of such diffusion, one French Guianese Creole man who is married to a Brazilian woman mentioned occasionally eating this species at home. Throughout Africa, where cassava has been important to many food systems since its introduction to the continent during the colonial period, cassava leaves feature prominently in many leafy dishes [56,57].

#### 2.2.12. Malvaceae

According to elderly people in our study and one historical reference (de Préfontaine [33]), the young leaves of *Hibiscus sabdariffa* L. (roselle or bissap in English) were once regularly added to French Guianese Creole dishes. In sub-Saharan Africa, the leaf, which is popular for its sour flavor, is a regular ingredient in sauce dishes [23]. Most notably, the leaves or calyxes are featured in the Senegalese dish *thieboudien* [58], an UNESCO world heritage dish [59]. The name *losey* (roselle) in French Guiana refers to the acidity of the leaves, which is similar to that of edible plants known as *oseille* (*Rumex* spp.) in France. In the French West Indies, the species is called ‘*grosey*’ in reference to the color of the products made with the calyxes, which are a bright red similar to that produced using currants and gooseberries in Europe (*Ribes* spp.; “*groseille*” in French).

In contrast, the leaves of the jute plant, *Corchorus olitorius* L., known as *lalo* in Haitian Creole, appears to be more specifically associated with the Haitian diasporic community. They are the main ingredient in the dish of the same name, which includes jute leaves cooked with pork and crab. This species seems to have been introduced to French Guiana by Haitian immigrants relatively recently and can now sometimes be found for sale in local farmers’ markets, especially in Cayenne. It can also be found in Haitian community shops in North America [60], where it appears to have been imported from West Africa. It is also an important leafy vegetable in many African countries, where it is widely cultivated and traded [61]. According to de Tussac [62], another species was probably used in the West Indies in the early 19th century, and was included in the Caribbean stew called *calalou*: *Corchorus siliquosus* L., an American species of the same botanical genus.

It seems that okra leaves, *Abelmoschus esculentus* (L.) Moench, known as *fey kalou* in French Guianese Creole, were also once eaten by French Guianese Creoles, as indicated by an elderly person from this community, but this practice appears to have disappeared today. Indeed, no okra leaves were found in local markets over the course of this study.

#### 2.2.13. Meliaceae

According to one respondent, neem buds, *Azadirachta indica* A. Juss., known as *lila peyi* in Haitian Creole, are consumed in Haiti. Since one Haitian woman in our study said that she consumed this plant, its use may have been introduced by the Haitian migrant community in French Guiana.

#### 2.2.14. Moringaceae

Based on our field observations and the comments we have gathered, *Moringa oleifera* Lam. leaves are sometimes cooked and eaten as a green by people from all communities, since this knowledge is widely shared on the internet. They can also be dried and ground into a powder used in soups and sauces. These culinary uses appear to be relatively recent in French Guiana, and have undoubtedly benefited from the increasing mediatic visibility of this species in recent years.

#### 2.2.15. Petiveriaceae

Two species of this family, both native to tropical America, were mentioned and are consumed exclusively by the Haitian community. *Rivina humilis* L., known as *panzou* or *lanman laye* in Haitian Creole, is cultivated for its leaves, although the red berries are also eaten. Another consumed species of this family is *Trichostigma octandrum* (L.) H. Walter, whose young leaves are eaten cooked by Haitian migrants. Its Haitian Creole vernacular name *lyann pannyé*, meaning “basket vine”, derives from the fact that the young branches were previously used to make baskets in the Greater Antilles [63]. Through processes of cultural exchange, this species is also increasingly consumed by French Guianese Creoles, although it remains strongly associated with Haitian communities, as reflected in its local designation as “Haitian spinach” (*zépina ayisyen* in French Guianese Créole).

#### 2.2.16. Phytolaccaceae

**Figure 7:**
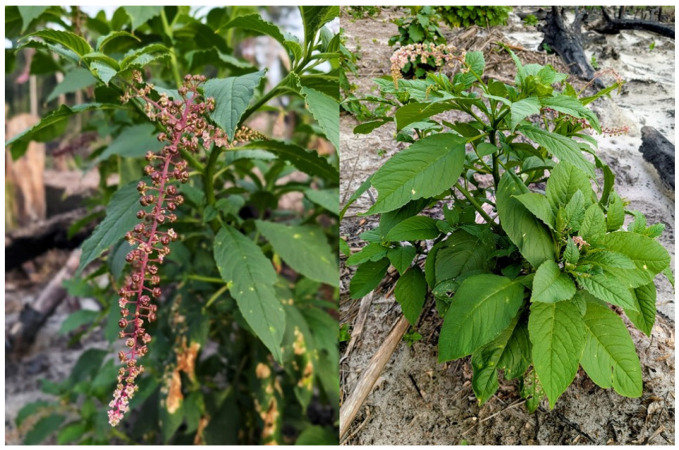
*Phytolacca thyrsiflora* specimens (fruits and leaves), photographed in a field in western French Guiana. Young leaves are consumed after cooking in French Guiana and Suriname. Photo credits: Marc-Alexandre Tareau, 2025.

*Phytolacca rivinoides* Kunth & C.D. Bouché is a wild Phytolaccaceae species native to South America. This species is called *bichoyak* in French Guianese Creole and *makoko* in Nengee Tongo and is often found growing wild in recently deforested or weedy areas and cassava fields. Young leaves are edible when cooked. Some interviewed participants specified that the leaves need to be boiled two or three times and the water discarded to remove the strong or “wild” taste. The leaves of *Phytolacca thyrsiflora* Fenzl ex J.A. Schmidt (Figure 7), which is also found in French Guiana, are consumed in parts of Brazil [64]. Given the similarity of this species to *P. rivinoides* (especially in the vegetative stage), it seems likely that it may also be consumed by Afro-descendants who mistake it for the more common *bichoyak*/*makoko*.

#### 2.2.17. Portulacaceae

Cultivated or foraged, *Portulaca oleracea*, known in Nengee Tongo as *poseen* (from the English word “purslane”, itself originating from the French word “*porcelaine*”) and *koupyé* in Creole (from the French name *pourpier*), is eaten by Creole communities. This species is not eaten by Maroons, who only use it for medicinal applications, including massage after maceration in coconut oil.

#### 2.2.18. Solanaceae

**Figure 8:**
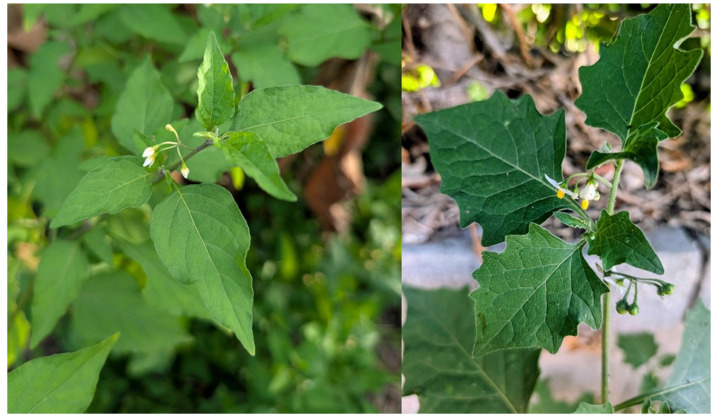
*Solanum americanum* (**left**) and *Solanum nigrum* (**right**; not found in FG). The leaves of both species are consumed after cooking. Note the distinctive lobed leaves of *S. nigrum*. Photo credits: Marc-Alexandre Tareau, 2025.

*Solanum americanum* Mill. (Figure 8), named *alaman* or *agouman* in French Guianese Creole, *lanman* in Haitian Creole and *(an)goma uwii* in Nengee Tongo, seems to have fallen into disuse in French Guiana, although it was widely eaten in the past. It was only cited as an edible plant by two Maroons and one Creole man, who said that he had not eaten it for decades. Nevertheless, it is widely sold in Surinamese markets and is still commonly consumed there, and is also eaten by Hmong communities in French Guiana (com. pers., A. Greene). The leaves of *Solanum* species are widely consumed in sub-Saharan Africa, particularly those of *Solanum aethiopicum* L. [65]. *Solanum macrocarpon* L. is cultivated in French Guiana for its fruits, but the leaves are not eaten, in contrast to what is observed in certain regions of the African continent [2].

*Cestrum latifolium* Lam., is also eaten by Maroon communities, who call it *bita uwii*, literally ‘bitter weed’. The species is sometimes confused with *Solanum leucocarpon* Dunal (*mananga* in Nengee Tongo and *mavévé chyen* in French Guianese Creole), which is however not consumed [66]. This species, like *S. americanum*, is absent from the markets of Cayenne; however, it can be found in the markets of Saint-Laurent-du-Maroni and in Suriname, where larger Maroon population are established. The distribution of its commercialization is thus a sign of the Maroon cultural affinity for this species.

One Ndjuka Maroon and one French Guianese Creole women said that the leaves of *Capsicum* spp. were edible, but specified that this was a very marginal use. Although their fruits are commonly consumed throughout the world, the consumption of chili pepper leaves as a cooked leafy vegetable has been documented in Korea [67,68], Japan [69], and Nigeria [70].

#### 2.2.19. Talinaceae

*Talinum paniculatum*, which is known as *caruru* in north Brazil [10] and *gran koupyé* or *koupyé Brésil* in French Guianese Créole, is eaten by Creole communities, who both cultivate and forage it. Maroons consider this plant akin to *Portulaca olearacea* and only use it for medicinal applications, including massage after maceration in coconut oil.

#### 2.2.20. Urticaceae

*Cecropia* spp. buds, *tronpèt* in Haitian Creole, are sometimes eaten by Haitians, according to one Haitian man interviewed during the survey. In addition, people we interviewed mentioned that *Laportea aestuans* (L.) leaves, called *zouti* in French Guianese Creole, were once eaten. However, since no one mentioned it during the survey, this use appears to have been lost today.

## 3. Discussion

### 3.1. Strong Cultural Preferences

To use an expression coined by Mintz and Price [71], Afro-descendant societies in the Americas are characterized by common symbolic containers but with different contents. Indeed, while the consumption of leafy vegetables (the container) seems to be anchored in the culinary practice of all the Afro-descent communities in our study, the specific plant used (the contents) varies widely from one population to another. These differences in the choice of species may sometimes be tinged with a multilateral ethnocentric distrust which, as is often the case in terms of food, gives rise to rejection or mockery of the practices of other groups. During our study, we often heard comments such as: “What, they eat that plant?! But that’s not something you eat!!”. Certain species are eaten exclusively by Maroons (*Cestrum latifolium*) and others by French Guianese (*Centropogon cornutus*, *Cucurbita maxima*, *Hibiscus sabdariffa*) or Haitian Creoles (*Cecropia* spp., *Corchorus olitorius*, *Rivina humilis*, *Trichostigma octandrum*). Some of these species can also be considered markers of cultural identity for each of these communities. A prime example is *C. olitorius*, of which one Haitian man we interviewed said: “*Tout koté i gen Ayisyen, ou jwenn lalo…* [Wherever there are Haitians, you’ll find *lalo* (*C. olitorius*).]”

We also noted differences between the communities in our study and Afro-descendant communities in other regions. For example, species of *Alternanthera*, *Celosia*, *Cleome* and *Hibiscus* are apparently no longer consumed in French Guiana, whereas they are still eaten by Afro-American communities in Brazil [10] and in northern Brazil [9]. The Asian species *Celosia argentea* L. and the African roselle, *Hibiscus sabdariffa*, both of which are consumed by Afro-descendant communities in other regions, are grown in French Guiana, but their leaves are not or only rarely consumed. These differences demonstrate the regional dynamism of Afro-descendant culinary traditions, likely in response to processes of cultural exchange with other communities. This finding also reinforces Mintz and Price’s idea that leafy greens as a cultural food group—the container—is highly resilient, while the species designated as leafy greens by individual Afro-descendant communities—the contents—varies over space and time.

### 3.2. Multiple Heritages, Highlighted by Vernacular Names

While the consumption of leafy vegetables is not a key element of the diets of Indigenous peoples in the Americas [9], this culinary practice is very popular in West Africa, Asia, and Europe. European settlers, enslaved Africans and Asian migrants brought this dietary preference (and a number of associated species) with them to French Guiana and Suriname, reshaping culinary practices in the region. Such processes of migration are not merely associated with the displacement of species, but also with the circulation, transformation, and the reconfiguration of knowledge and practices. In this context, the persistence and evolution of leafy vegetable consumption can be understood as part of broader processes of interculturization, through which ethnobotanical knowledge is continuously reshaped through interactions between diverse cultural groups [72].

Vernacular names recount this story of knowledge transfer and migration. The diversity of vernacular names attributed to certain species points to their cultural transversality, revealing their circulation across different cultural groups. Conversely, species for which only a single vernacular name was recorded tend to reflect more culturally specific patterns of use.

The integration of the French words “*épinard*”, “*choux*”, “*oseille*”, and “*brède*”, meaning respectively spinach, cabbage, sorrel and edible leaves, into local Creole languages shows the interest Europeans took in edible leaves and their tendency to transfer the names of European species to analogous tropical plants. The use of the terms “*choux de Chine*” and “*choux chinois*” (Chinese cabbage) to refer to species introduced from Asia to the Americas reveals a similar logic of combining a European folk taxon (*chou*) with a geographical indicator. The relatively ancient introduction of edible Asian leaves to the Guianas continues today through the Hmong community [73], who also consume other species of leafy greens not currently used by Afro-descendant communities (i.e., *Alternanthera* spp., *Talinum* spp., *Gynura* spp. and *Eupatorium ayapana* Vent.). We can assume that over time, these culinary habits will spread to other cultural communities in French Guiana. Indeed, since the Hmong community are the main agricultural producers in French Guiana, the plants they grow primarily for their own consumption, but which are also sold at markets, are likely to gradually spread to other cultural groups. In cases where the Hmong already consume the same leafy greens as local populations, this concurrence may reinforce the continued use of these species, preventing their loss to cultural entropy.

Other plant names reflect the African origin of some ethnobotanical knowledge. For example, *Solanum americanum* is sometimes called *agouman* by Creoles, as well as *angoma uwii* and *goma wiri* by Maroons. These terms come from the African taxon *gboma* which, in the Fon and Ewe languages, refers to *Solanum aethiopicum* L. [74]. Similarly, the Ndjuka Maroon ethnotaxon *mboya* for *A. blitum* likely originates from the term *imbuya*, which refers to amaranth leaves in certain Bantu languages [75]. These examples show that African ethnobotanical and lexical knowledge was successfully transferred by enslaved Africans to the Guianas, and that traces of this African knowledge persist today in the knowledge traditions of Afro-descendant populations in the region.

The French Guianese Creole term *kalalou* is also extremely revealing of the complex history of interculturalization linked to slavery. Indeed, we find this term and its derivatives (*kalou*, *calalou*, *cariru*, *callaloo*, etc.) in nearly all African American communities, where it designates edible plants or dishes made from leaves. The ubiquity of this term could be explained by its diffusion at the same time as the importation of slaves throughout the Americas. In Haiti, *calalou* is used for *Abelmoschus esculentus* and *lalo* for *Corchorus olitorius*. In Jamaica, *callaloo* refers to *Amaranthus viridis* or *Hibiscus sabdariffa* [76]. However, Higman [24] notes that in Jamaica there is also a “mountain callaloo” (*Phytolacca rivinoides*), a “Spanish callaloo” (*Amaranthus dubius*), a “toyer callalloo” (*Xanthosoma* sp.) and a “branched callaloo” (*Solanum* sp.). In the Caribbean coast of Panama, *calalú* or *kalalu* refers to the fiddleheads of edible ferns, likely those of *Thelypteris* spp. (com. pers., A. Greene).

**Figure 9:**
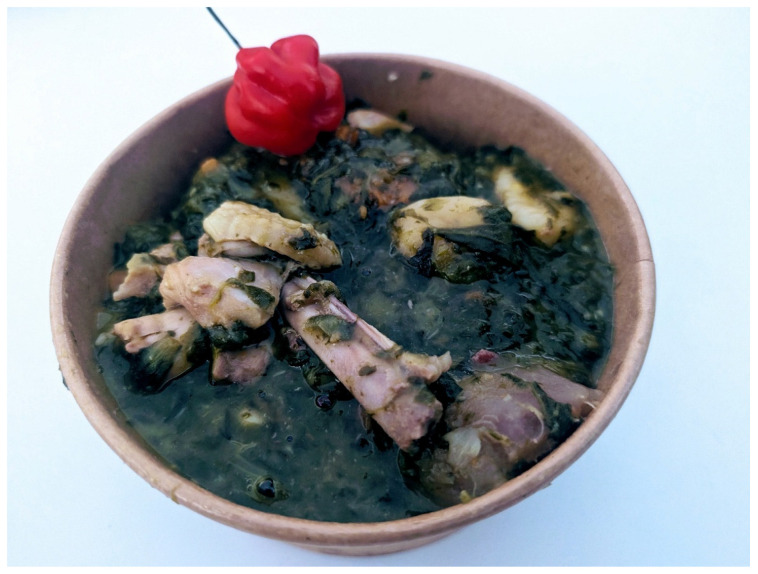
A bowl of *lasoup zabitan*, which is a local variant of the widespread Caribbean dish callaloo, made primarily with *Basella alba* leaves at a food stall in Cayenne (French Guiana). Photo credit: Michael Rapinski, 2025.

In Brazil, several amaranth species are called *carurú* [77] while *carurú-azedo* refers to *Hibiscus sabdariffa* [78], and *carurú-cipó* to *Basella alba* [10]. For adherents of the Afro-Brazilian religion Candomblé, jute mallow is known as *carurú-da-Bahia*. In other parts of Brazil, *cariru* corresponds to *Talinum* spp. [9], while on the Rio Negro, *Basella alba* and *Phytolacca rivinoides* are called *caruru*. In French Guiana, the Creole term *kalou* refers to *Abelmoschus esculentus* while Maroons use *kalalu* for *Amaranthus dubius* and *kalu* for corn (probably the only case where the word does not refer to a leafy vegetable). In the French Antilles, *calalou*/*kalalou* (or “*herbes à calalou*”) refers to leaves of *Xanthosoma* and *Colocasia* species [79]. Finally, in Louisiana and throughout the Caribbean, *callaloo*/*kalalou*/*calalù*/*kalou* also refers to a characteristic dish whose main ingredient is okra [80], but which also contains leafy vegetables and meat, fish or crab. When this leafy stew is made without okra and is therefore much less viscous, or even not viscous at all, it is called *lasoup zabitan* (Figure 9). in French Guianese Creole, or *lasoup vèrt* (“green soup”, because the leaves are generally blended more than in the classic *kalalou*).

Several sources indicate that the term *kalalou* has its linguistic origins in Africa [77,81]. Voeks [77], in particular, maintains that it is a word from the Kimbundu language that refers to several edible plants in Central Africa, particularly those of the genus *Vernonia*. However, it has also been suggested that *kalalou* may be a word of Native American origin [24]. According to Père Breton [32], for instance, *calao* is a Carib word referring to a type of potherb. It could also originate from the Tupi-Guarani term *caàrurù* [77,81], meaning “herbaceous plant” (com. pers., Pierre and Françoise Grenand). In her doctoral thesis, the linguist Elodie Jourdain [82] argued that the term probably originated from an Amerindian word that was borrowed by the Portuguese, taken to Africa and then brought back to the Americas by enslaved peoples. This theory would explain why cognates are found on both sides of the Atlantic Ocean. For example, in Angola and São Tomé and Príncipe, two Portuguese-speaking countries in Africa, *calalou* is considered the national dish.

However, cognate terms are found much more widely in Africa and exist across many language families. In Senegal, the Fulani, Peul and Wolof peoples use the term *lalo* (or *laalo*) for all green leafy vegetables that have a mucilaginous texture and can be used as a binding agent in millet couscous, including baobab (*Adansonia digitata* L.), okra (*Abelmoschus esculentus* L.), jute mallow (*Corchorus olitorius* L.), *bissap* (*Hibiscus sabdariffa* L.) and *gum karaya* (*Sterculia setigera* Delile) leaves [58,83,84]. The Fulani of northern Cameroon apply the term *lalo tile* to *Corchorus olitorius* L. and *lalo* to another unspecified *Corchorus* sp. [85], a striking parallel with Haitian Creoles, who use the exact same term for this plant. The Moors of Mauritania also use the terms *lalo trab* and *lalo* for the horn-fruited jute (*Corchorus tridens* L.) [86]. The Toubou of the central Sahara use *kolou* for this plant, whose leaves are prepared in sauces [87]. Could the diversity of such terms provide evidence of the African origin hypothesis of *kalalou*? Or did pastoralists and trading merchants contribute to the rapid dispersal of its cognates around the greater Sahara region?

We agree with Voeks [77] that the enigma posed by the etymology of the word *kalalou* is an incredible puzzle whose historical origin is less straightforward than generally claimed in the literature. Although we are not linguists, we tend to believe that the term probably emerged in the Americas during processes of linguistic creolization. Such creolization may have resulted in the hybridization of the Tupi-Guarani term *caàrurù* with the West-African word *laalo*. Since *caàrurù* refers to various species of American amaranths introduced to Africa during colonization [88,89], and *laalo* refers to viscous edible plants, the terms could have easily become conflated during trans-Atlantic flows of people, knowledge and plants. In this case, both terms may have crossed the Atlantic, with *caàrurù* traveling east to Africa and *laalo* traveling west to the Americas. As an example of the latter, the term *loli*, meaning “slimy” in the Ndjuka Maroon language of French Guiana and Suriname (Personal communication with Clarisse Ansoe-Tareau, 23 May 2026), functions as a descriptive attribute rather than a botanical identifier, yet it hints at a possible linguistic derivation from the West African *laalo*.

### 3.3. Evolving Culinary Habits

This study highlights the dynamism of culinary practices, which evolve considerably over time. Recently introduced species are gradually being adopted into the diets of local populations, while others remain in use. For example, species such as *P. rivinoides*, *C. latifolium*, and *C. esculenta* were already reported as being consumed by Maroon communities in the inventories of Fleury [17] and Bilby et al. [18], indicating a long-standing tradition of consumption, whereas the use of some previously consumed species is declining. Among the Creoles of French Guiana, for example, the consumption of the native species *Phytolacca rivinoides*, which seems to have been common in the past, has nearly disappeared. Both Chevalier de Préfontaine [33] and Dr. Sagot [90] note the importance of this plant in the French Guianese diet, specifying that it is known as *épinard* (spinach) and is commonly cooked like spinach. Today, this species has been eclipsed in the French Guianese diet by Asian species like *Basella alba* and *Brassica* spp., which are now widespread in farmers markets. The Inventory of culinary heritage in France also notes that *Phytolacca rivinoides* appears to have been the main type of ‘spinach’ in French Guiana in the past before gradually being replaced by *Basella alba* [35].

This change undoubtedly reflects the influence of the Hmong community, which has come to dominate agriculture in French Guiana and cultivates and consumes many Asian leafy greens [91]. Hmong farmers may also be playing a role in the increasing prominence of other species, including *Ipomoea aquatica*, *Ipomoea batatas* leaves and *Amaranthus* spp. Such shifts in the availability and consumption of leafy greens highlight how migration flows reshape the agrobiodiversity of local food systems. Although such changes may contribute to increasing food security by stabilizing the availability of local food resources, they may also lead to decreases in culturally significant foods that could play a role in food sovereignty and food system resilience.

Other examples of species that seem to have fallen into disuse in French Guiana include *Centropogon cornutus*, *Laportea aestuans* and *Solanum americanum,* all of which grow spontaneously (Table 1). Some elderly people interviewed during this study speak with nostalgia about eating the leaves of these plants during their youth:

“*Anvan nou té ka mété bokou radyé annan nou manjé. Bichoyak, zèb anmè, mouzanbé… Tou sa a bagaj ki disparèt jòdi jou. Jenn yan pa konnet sa zerb ya ankò*… [We used to add lots of herbs to our dishes. *Bichoyak (P. rivinoides)*, *zeb anmè* (*S. americanum*), *mouzambé (C. gynandra)*… All of these herbs have disappeared today. Young people no longer recognize these herbs.]”

French Guianese woman living in Matoury.

Nevertheless, this decrease in interest in certain species is partially offset by substitute plants brought to the territory by recent migrants [92]. For instance, our results indicate that Haitian migrants have recently introduced *Corchorus olitorius* to French Guiana. Similarly, Brazilian farmers cultivate *Acmella oleracea*, *Brassica oleracea* and *Talinum paniculatum*, contributing to the spread of these species and their adoption by other cultural communities.

However, when we compare our results with those found in historical literature, we observe a clear decline in the variety of leafy greens consumed in French Guiana. Consider, for example, this recipe for a *calalou* dish transcribed by Descourtilz [34] (p. 5):

*“We made an excellent calalou with “chou caraïbes” leaves, small spiny cucumbers from the savannahs, sweet spinach, and young pumpkin shoots, okra cones, kaia mouzambai foliage* [*Cleome gynandra*?]*, nightshade, purslane, melon buds, oxalis* [*Oxalys barrelieri*?]*, roselle, small valeriane with red flowers and silver undersides, commonly known as patagon* [*Boerhavia* sp.?]*, tomatoes, tender potato leaves, and chili peppers.”* (Translation by the authors of the following original quotation: “*On compose un excellent* calalou *(mets créole) avec les feuilles du Chou Caraïbes, le petit concombre épineux des savanes, les épinards doux et les jeunes pousses de giromon, les cônes du gombo, le feuillage du kaia mouzambai, la morelle, le pourpier, les bourgeons de melon, l’oxalide, l’oseille de Guinée, la petite valériane à fleurs rouges et à feuilles argentées en dessous, vulgairement appelée patagon; les tomates, feuilles tendres de patates et les pimens*”.)

Today, this same dish generally contains okra and *Basella alba* as the only green vegetables, highlighting a significant loss of ingredient diversity over the past 200 years.

Our finding that many species of leafy greens are in the process of being lost supports the conclusion of Vandebroek and Voeks [10], who have argued that the use of African vegetables is gradually fading away across Afro-descendant populations in the Americas. This trend raises important concerns about the sustainability and integrity of local food systems, as the loss of dietary diversity can undermine the nutritional adequacy of diets [93,94,95,96,97,98] and overall well-being [99,100,101]. Local policymakers in French Guiana and Suriname could help reverse this trend by working to safeguard food system diversity. For instance, the creation of policies promoting the use of traditional leafy greens and dishes like *calalou* would support the maintenance of culinary heritage while also reinforcing regional food security.

It seems possible that the recent trend for “Non-conventional food plants” [102], as many of the species described in this study are sometimes called, could contribute to reversing the trend of diminishing culinary diversity. The increasingly globalized access to information about food plants through social media and online sources could also revive some of these species and bring new ones to the fore. The examples of *Moringa oleifera* and *Leuenbergeria bleo*, both of which were described by interviewees as having been learned about online, demonstrate the growing influence of the digital sphere on the transformation of cultural practices and dietary trends.

Beyond their cultural and culinary significance, the diversity of leafy vegetables documented in this study also has broader environmental implications. Recent research has highlighted the important role of Afro-descendant communities in shaping biodiverse and resilient landscapes across tropical regions of South America [103]. In particular, Afro-descendant lands have been shown to coincide with areas of high biodiversity and carbon storage, and to be associated with 29–55% lower deforestation rates compared to surrounding areas [103]. These patterns have been linked to long-standing land management practices rooted in African-derived ecological knowledge and adaptive strategies in tropical environments.

The importance of diversified and locally adapted food resources has also been emphasized in recent global analyses showing that countries worldwide remain highly dependent on external food systems and vulnerable to supply disruptions [104]. However, Guyana, located within the Guiana Shield region, is the only country to achieve food self-sufficiency in the seven food groups of the Livewell diet, including vegetables, through domestic production [104]. The potential contribution of non-conventional leafy vegetable foods to such self-sufficiency remains to be evaluated. Nonetheless, the diversity of leafy vegetables, such as those highlighted in the present study, may be understood not only as a reflection of cultural heritage and intercultural exchange, but also as part of broader socio-ecological systems that contribute to the maintenance of agrobiodiversity and food security. The continued use and transmission of such plant knowledge may therefore play a role in supporting more diverse and resilient food systems, with potential relevance for sustainability-oriented approaches to plant–human interactions.

### 3.4. Risk Perception and Colonial Legacies

Divergent opinions on the toxicity of certain plants appeared during our study: some communities consider a species to be toxic, while others consider it to be edible and healthy. *Phytolacca rivinoides* presents a peculiar case; although respondents claim that it is safe to eat, the reported practices of boiling the leaves in successive changes in water before consumption suggests a tacit recognition of potential toxicity. This may be understood as a culturally embedded risk management strategy, allowing the plant to remain socially classified as edible while acknowledging the need for careful preparation to minimize perceived danger. Indeed, a class of compounds associated with cytotoxic activity has been identified in this [105] and other *Phytolacca* spp. [106]. Moreover, *P. rivinoides* is frequently confused with the American pokeweed (*P. americana* L.) whose poisonous reputation precedes it due to the occurrence of pokeweed mitogen and triterpenoid saponins [107,108]. Traditional practices advise boiling the leaves of *P. americana* in at least 2–3 changes in water to reduce toxicity before they can be eaten [35].

*Solanum americanum* Mill., however, best exemplifies divergent risk perceptions. Creole people in French Guiana have stopped consuming it due to its reputation as a toxic plant, whereas Maroons continue to eat it. This “bad reputation” is due to its similarity with the closely related *Solanum nigrum* L. (Figure 8), which does not occur in the Guianas. *S. nigrum* is called “black nightshade” [22] and is widely reported to be toxic due to the presence of glycoalkaloids [109,110]. However, the amount of these compounds gradually decreases with plant growth and maturity [110], and evidence for their presence in *S. americanum* is limited and contradictory [111,112,113,114]. In any case, the leaves of species within the *S. nigrum* complex, including *S. americanum* [115], are widely consumed as a food and medicine without apparent harm by cultures throughout Africa and Asia [109,110,115,116,117,118]. Some studies argue that the toxicity of these species is nonexistent or overstated [114,118], a conclusion that would validate Maroon traditional knowledge of *S. americanum*.

In that case, where has the toxic reputation for this common potherb come from? By the mid-15th century, European *Solanum* spp. such as black nightshade were already being conflated with deadly nightshade (*Atropa belladonna* L.), whose similar-looking berries contain highly toxic tropane alkaloids such as atropine and scopolamine [118]. The attribution of poisonous properties to *Solanum americanum* thus appears to be a misunderstanding imported from Europe, and its presence among Creole populations in the Guianas reflects a colonial inheritance. The labeling of this common plant as toxic, a belief shaped by European taxonomic confusion, may have effectively marginalized Afro-descendant knowledge regarding the safe culinary use of *S. americanum*. This marginalization has contributed to enduring narratives of toxicity connected to food practices associated with the transatlantic slave diaspora and has left a lasting negative impact on local food security and sovereignty.

## 4. Materials and Methods

### 4.1. Ethnographic Background

**Figure 10:**
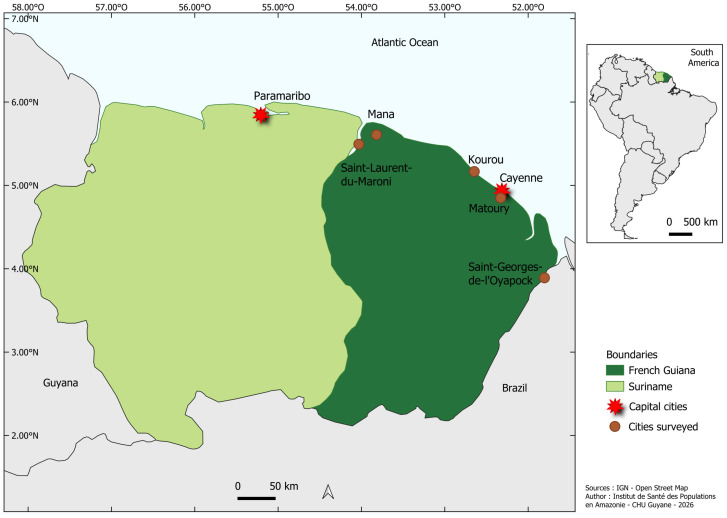
Map of study area showing the location of cities where surveys were conducted in French Guiana and Suriname.

In addition to the French Guianese and Surinamese Creoles and Maroon groups who have historically been present in the region [119], the Haitian migrant community is increasingly numerous in French Guiana [120]. Their numbers in French Guiana are estimated at 50,000 out of a total population of 300,000 [121], particularly in Cayenne and westward on towards the Surinamese boarder (Figure 10). Their inclusion in this survey enabled a comprehensive inventory of the leafy vegetables eaten by different cultural groups of African descent living in the French Amazon. Furthermore, French Guianese Creole and Maroon groups are present throughout the entire coastal region of French Guiana, whereas Surinamese Creole and Ndjuka (here referring to the only Maroon group represented in this study) are primarily present in Suriname and western French Guiana up to Cayenne. The population of Suriname is estimated at 635,000 inhabitants [122].

This ethnobotanical study adopts a deliberately descriptive approach with the aim of documenting the diversity of plant uses and practices across cultural groups. Such an approach is particularly relevant in contexts where baseline data remain scarce and where documenting variability constitutes a necessary first step before further analytical generalization.

### 4.2. Interviews

This study employs an ethnographic approach combining field observations and informal and semi-structured interviews to produce qualitative data on the consumption of leafy green vegetables. Participants were identified through purposive and snowball sampling using local community networks. Interviews were conducted primarily in participants’ everyday living environments in order to capture practices within their social and cultural contexts [123]. Data collection continued until reaching theoretical saturation, when no substantially new information or species emerged from additional interviews [123].

Informal interviews (N = 26) were conducted with knowledgeable people (plant vendors, traditional healers, farmers, cooks) from four distinct cultural groups, namely Haitians, French Guianese Creole, Surinamese Creole and Ndjuka (Table 2).

These included garden (*n* = 7) and markets (*n* = 5) surveys in Cayenne, Kourou, Mana, Matoury, Saint-Georges-de-l’Oyapock, Saint-Laurent-du-Maroni (French Guiana) as well as Paramaribo (Suriname) (Figure 10 and Table 2), following the methodology described by Vogl et al. [124]. Interviews were conducted in these same cities by the authors in French, French Guianese Creole, Haitian Creole, Nengee Tongo, and Sranan Tongo. Nengee Tongo is the language spoken by Aluku, Okanisi/Ndjuka and Paamaka Maroon groups, whereas Sranan Tongo, meaning ‘Surinamese tongue’, is the *lingua franca* in Suriname. Originating as an English pidgin in the 17th century, Sranan Tongo has evolved to include significant vocabulary and grammatical influence from Dutch, Portuguese, and various African and Indigenous languages [119].

Interviews were structured around two simple guiding questions: “Do you eat edible leaves?” and “If so, which ones?”, followed by open-ended questions addressing preparation methods, uses, and cultural significance. Data were recorded through field notes. Given these exploratory methods, the inventory presented in this study should be understood as a documented snapshot of edible leafy plants mentioned or observed during fieldwork rather than an exhaustive account of all species used in the study area. Survey data were then supplemented by reviewing historical and botanical works that mention the utilized species to compare past uses with those observed today.

### 4.3. Limitations of the Study

This study presents several limitations inherent to its qualitative and exploratory design. First, the sample size (N = 26) does not aim at statistical representativeness but rather at capturing the diversity and depth of ethnobotanical knowledge. The composition of the sample may also reflect certain biases, particularly in terms of age, gender, and level of expertise, as participants were selected based on their recognized knowledge of plant uses rather than demographic attributes. In addition, the representation of cultural groups is not strictly balanced, and the high level of cultural diversity in the study area means that only a limited number of individuals could be interviewed within each group, which may influence the relative visibility of certain practices over others.

Furthermore, the reliance on informal and semi-structured interviews may have led to variability in the type and depth of information collected. However, this is also consistent with a flexible, ethnographic approach that allows participants to express their knowledge on their own terms and facilitates the emergence of nuanced and context-dependent information. These limitations should therefore be understood in light of the study’s objective, which is to explore patterns of diversity, circulation, and transformation of plant-based practices rather than to provide a quantitative or exhaustive survey.

### 4.4. Plant Identification

No botanical vouchers were made of the species cited in this paper. Instead, all species mentioned were photographed and identified using the photographic voucher identification methodology proposed by Greene et al. [125]. Identified species were cross-referenced with existing botanical vouchers from the Cayenne Herbarium to confirm taxonomic identifications. A few species that could not be cross-checked with existing herbarium vouchers are well-known plants in the study area that do not present identification challenges. Plant species identifications were further validated by external botanists familiar with the regional flora. Current scientific names and families were verified using the Taxonomic Name Resolution Service (TNRS) [126,127], which relies on the World Flora Online (WFO) and World Checklist of Vascular Plants (WCVP) taxonomic backbones. Names were also cross-checked with TAXREF [128,129], the national taxonomic reference used by the French National Inventory of Natural Heritage (INPN). The native or introduced status of plant species in French Guiana and Suriname was determined using Plants of the World Online [22] and TAXREF.

In this study, we focused exclusively on leaves that are consumed cooked. Leaves eaten raw, which play a very limited role in local food systems, were not inventoried, nor were those used as herbs and spices, since the authors consider these to belong to distinct use categories. All vernacular names are given in italics. It should be noted that, while the vernacular names mentioned in the results (Table 2) may suggest the cultural groups and communities that consume these species, the language in which a plant name is cited cannot be exclusively attributed to a single cultural group. The cultural landscape of French Guiana and Suriname is characterized by considerable interactions between communities, with many individuals identifying with multiple heritages and cultural backgrounds. Indeed, despite the languages in which interviews were conducted, some plant names were provided in multiple languages, including those other than the primary language of communication. As a result of complex and dynamic individual identities, it is not always possible to associate specific ethno-culinary knowledge or practices with a single cultural group. Vernacular names were therefore provided with their corresponding languages in Table 1 without explicit assignment to a specific cultural group. The ecological status of the species surveyed is indicated by the abbreviations **C** for cultivated and **W** for wild.

## 5. Conclusions

The diversity of leafy vegetables consumed by Afro-descendant communities in French Guiana and Suriname reveals how a wealth of traditional knowledge contributes to the deeply dynamic nature of food practices. Far from being fixed, culinary habits are constantly shifting: some species fall into disuse, while others are reintroduced or adopted. Leafy greens form part of a living, adaptive repertoire that reflects the ecological context, social transformations, and cultural negotiations of each cultural group, all of which contribute to processes of dietary transformations in this rich, multicultural region.

The term *calalou* (and its linguistic variants across the Americas) offers a powerful metaphor for this fluidity. Like the dish it names, *calalou* brings together different elements from various origins, simmered into a coherent whole. Similarly, the Afro-descendant ethno-culinary repertoire in the Guiana Shield is the result of multiple heritages: African, Amerindian, European, Asian, and contemporary global influences. This interculturalization process [72] is visible both in the names of plants and in the ways they are prepared, used, adopted, or rejected by different cultural groups. Since “callaloo” is such a powerful symbol of multiculturalism and Creole societies, we could call this a process of “callalooization” [130].

Overall, our findings highlight three main patterns: (1) a significant diversity of edible leafy species used across Afro-descendant communities in the Guianas, (2) marked differences in species selection, uses, and meanings between cultural groups, and (3) both continuity and transformation of plant-based practices linked to African and Afro-American traditions. These results open several avenues for future research. Further studies could explore the nutritional properties of these underutilized leafy vegetables, as well as the processes of intergenerational transmission of ethnobotanical knowledge, particularly in the context of rapid social change. In addition, greater attention could be given to the role of these plants in food security, public health, and policy-making, especially in relation to the valorization of local and culturally significant food resources.

Finally, these leafy edible greens are unquestionably more than ingredients; they are markers of identity, memory, and health. Their consumption is often accompanied by narratives of resilience, strength, and cleansing, thus explicitly positioning them on the food-medicine continuum. Our study underscores the need for further research using quantitative methods to assess the circulation, valuation, and transmission of plant-based knowledge within and across Afro-descendant communities. Situating these practices within the broader socio-economic context of food system transformation emphasizes the role of food agency in building resilient, secure and sovereign food systems. Furthermore, exploring these culinary trajectories can help reframe leafy vegetables not merely as nutritional resources and agrobiodiversity markers, but also as cultural and historical archives: green traces of a long journey through colonization, resistance, and transformation.

## Figures and Tables

**Table 1:** Families and species of leafy green vegetables cited as a food during ethnobotanical surveys in French Guiana and Suriname. Vernacular names are given with language codes (BR = Brazilian Portuguese; FGC = French Guianese Creole; HC = Haitian Creole; NT = Nengee Tongo; SRT = Sranan Tongo). Source indicates whether the plant is cultivated (C) or wild (W); status indicates native (N), introduced (I) or no information (NI), country indicates French Guiana (FG) and Suriname (SR).

Family *Species*	Vernacular Names	Source	Status ^1^	Range ^2^	Sold in Markets	Country
**Amaranthaceae**						
*Amaranthus blitum* L.	*ti zepina (peyi)* (FGC, HC), *kalalu*, *mboya* (NT)**,** *klaroen* (SRT)	W	I	SA	Yes	FG; SR
*Amaranthus dubius* Mart. ex Thell.	*zepina (peyi)*, *zergon* (FGC, HC), *kalalu* (NT)**,** *klaroen* (SRT)	W/C	I*	TA	Yes	FG; SR
*Amaranthus cruentus* L.	*zepina (peyi)* (FGC, HC), *kalalu* (NT)**,** *klaroen* (SRT)	W/C	I	CA	Yes	FG; SR
*Amaranthus spinosus* L.	*zepina kochon* (FGC, HC)	W	I	TA	No	FG
**Araceae**						
*Colocasia esculenta* (L.) Schott	*fey dachin* (FGC), *fey mazonbel* (HC)**,** *tajer blad* (SRT)**,** *sineisi taya uwii* (NT)	C	I	SEA	Yes	FG; SR
*Xanthosoma brasiliense* (Desf.) Engl.	*buter blad* (SRT), *zerbaj* (FGC)	C	NI	TA	Yes	FG; SR
*Xanthosoma sagittifolium* (L.) Schott	*fey tayov*, *djoubéka* (FGC), *fey malanga* (HC), *taya uwii* (NT), *tajer blad* (SRT)	C	I	TA	Yes	FG; SR
**Asteraceae**						
*Acmella oleracea* (L.) R.K.Jansen	*flèr maldan*, *kréson Parà* (FGC), *jambu* (BR)	C	I	SA	Yes	FG
*Bidens* spp.	*zedjwi dyab* (HC)	W/C	N	Cosm.	No	FG
**Baselaceae**						
*Basella alba* L. (syn: *Basella rubra* L.)	*zepina* (FGC, HC)**,** *spenasi* (NT, SRT)	C	I	SEA	Yes	FG; SR
**Brassicaceae**						
*Brassica juncea* (L.) Czern.	*amsoi*, *chap soi*, *fey chou chinwa* (FGC, SRT, NT)	C	I	CEuA	Yes	FG; SR
*Brassica rapa* var. *chinensis* (L.) Kitam.	*pakchoi*, *bok choi*, *fey chou chinwa* (FGC, SRT, NT)	C	I	A, M	Yes	FG; SR
*Brassica oleracea* L.	*fey chou* (FGC), *kale* (BR)	C	I	E	Yes	FG
**Cactaceae**						
*Leuenbergeria bleo* (Kunth) Lodé.	*fey pereskya* (FGC)	C	I	TA	No	FG
**Campanulaceae**						
*Centropogon cornutus* (L.) Druce	*radyé pété* (FGC)	W	N	TA	No	FG
**Cleomaceae**						
*Cleome gynandra* L.	*mouzambé* (FGC)	W	I	A, IS	No	FG
**Convolvulaceae**						
*Ipomoea aquatica* Forssk.	*lizron dlo* (FGC)**,** *dagoe blad* (SRT)	C	I	A, SEA	Yes	FG; SR
*Ipomoea batatas* (L.) Lam.	*fey patat* (FGC, HC)	C	I	TA	Yes	FG
***Cucurbitaceae***						
*Cucurbita maxima* Duchesne	*fey jomou* (FGC, HC), *pampun uwii* (NT)	C	NI	SA	No	FG; SR
*Luffa* spp.	*fey konkonm tòrchon* (FGC, HC)	C	N	Cosm.	No	FG
*Sicyos edulis* Jacq.	*fey kristofin* (FGC), *fey chayòt* (HC)	C	I	CA	No	FG
**Euphorbiaceae**						
*Manihot esculenta* Crantz	*maniva* (BR), *fey mannyòk* (FGC, HC)	C	N	SA	Yes	FG
**Malvaceae**						
*Abelmoschus esculentus* (L.) Moench	*fey kalou* (FGC), *fey kalalou* (HC), *okoo uwii* (NT)	C	I	IS	No	FG; SR
*Hibiscus sabdariffa* L.	*fey lozey* (*peyi*) (FGC)	C	I	A	Yes	FG
*Corchorus olitorius* L.	*lalo* (HC)	C	I	A, IS	Yes	FG
**Meliaceae**						
*Azadirachta indica* A.Juss.	*fey lila*, *lila peyi* (HC)	C	I	SEA	No	FG
**Moringaceae**						
*Moringa oleifera* Lam.	*morenga* (FGC), *benzoliv* (HC)	C	I	IS	No	FG
**Petiveriaceae**						
*Trichostigma octandrum* (L.) H.Walter	*lyann pannyé* (HC), zépina ayisyen (FGC)	W/C	N	TA	Yes	FG
*Rivina humilis* L.	*panzou*, *lanman layé* (HC)	C	I*	TA	Yes	FG
**Phytolaccaceae**						
*Phytolacca rivinoides* Kunth & C.D. Bouché	*bichoyak* (FGC), *makoko* (NT)	W	N	TA	No	FG; SR
**Portulacaceae**						
*Portulaca oleracea* L.	*ti koupyé* (FGC, HC), *poseen* (NT)	W/C	I	A, EuA	Yes	FG
**Solanaceae**						
*Capsicum* spp.	*fey piman* (FGC, HC)**,** *pepee* (NT)	C	I*	TA	No	FG; SR
*Cestrum latifolium* Lam.	*bita uwii* (NT)**,** *bita wiri* (SRT)	W/C	N	TA	Yes	FG; SR
*Solanum americanum* Mill.	*agouman*, *alaman* (FGC), *angoma uwii* (NT)**,** *lanman* (HC), *goma wiri* (SRT)	W/C	I*	Am	Yes	FG; SR
**Talinaceae**						
*Talinum paniculatum* (Jacq.) Gaertn.	*gran pourpyé* (FGC, HC)	W/C	N	TA	Yes	FG
**Urticaceae**						
*Laportea aestuans* (L.) Chew	*zouti* (FGC, HC)	W	N	TA, A, IS	No	FG
*Cecropia* spp.	*fèy tronpèt* (HC)	W	N	TA	No	FG

1. I* = Conflicting information between POWO and TAXREF. 2. A = Africa; Am = Americas; CA = Central America; CEuA = Central Eurasia; E = Europe; EuA = Eurasia; IS = Indian Subcontinent; M = Mediterranean; SA = South America; SEA: Southeast Asia; TA = Tropical America; Cosm. = Cosmopolitain.

**Table 2:** Distribution of interviewees across cultural groups, including number of participants, gender ratio, study locations and languages spoken.

Cultural Groups	Number of Interviewees	Sex Ratio ^1^	Locations	Languages
Ndjuka	9	F: 6; M: 3	Mana, Paramaribo, Saint-Laurent-du-Maroni	Nengee Tongo, Sranan Tongo
French Guianese Creoles	9	F: 7; M: 2	Cayenne, Kourou, Matoury, Saint-Georges-de-l’Oyapock	French Guianese Creole, French
Haitian Creoles	6	F: 3; M: 3	Cayenne, Kourou, Matoury, Saint-Laurent-du-Maroni	Haitian Creole, French
Surinamese Creole	2	F: 1; M: 1	Paramaribo	Sranan Tongo

^1^ F = Female, M = Male.

## Data Availability

Data are available from the corresponding author upon reasonable request.
